# Spiroketones and a Biphenyl Analog from Stems and Leaves of *Larrea nitida* and Their Inhibitory Activity against IL-6 Production

**DOI:** 10.3390/molecules23020302

**Published:** 2018-01-31

**Authors:** Jongmin Ahn, Yihua Pei, Hee-Sung Chae, Seong-Hwan Kim, Young-Mi Kim, Young Hee Choi, Joongku Lee, Minsun Chang, Yun Seon Song, Roberto Rodriguez, Dong-Chan Oh, Jinwoong Kim, Sangho Choi, Sang Hoon Joo, Young-Won Chin

**Affiliations:** 1 College of Pharmacy and Research Institute of Pharmaceutical Sciences, Seoul National University, Seoul 08826, Korea; jm212224@snu.ac.kr (J.A.); yanberk@hanmail.net (S.-H.K.); dongchanoh@snu.ac.kr (D.-C.O.); jwkim@snu.ac.kr (J.K.); 2 College of Pharmacy and Integrated Research Institute for Drug Development, Dongguk University-Seoul, Gyeonggi-do 10326, Korea; yeahhwa@gmail.com (Y.P.); chaeheesung83@gmail.com (H.-S.C.); 0210121@hanmail.net (Y.-M.K.); choiyh@dongguk.edu (Y.H.C.); 3 Department of Environment and Forest Resources, College of Agriculture and Life Sciences, Chungnam National University, Daejeon 34134, Korea; joongku@cnu.ac.kr; 4 Department of Biological Sciences, College of Science, Sookmyung Women’s University, Seoul 04310, Korea; minsunchang@sookmyung.ac.kr; 5 College of Pharmacy, Sookmyung Women's University, Seoul 04310, Korea; yssong@sookmyung.ac.kr; 6 Department of Botany, University of Concepcion, Casilla 160C, Concepcion 4080871, Chile; rrodrigu@udec.cl; 7 International Biological Material Research Center, KRIBB, Daejeon 34141, Korea; decoy0@kribb.re.kr; 8 College of Pharmacy, Daegu Catholic University, Gyeongbuk 38430, Korea; sjoo@cu.ac.kr

**Keywords:** *Larrea nitida*, spiroketones, biphenyl analog, HMC-1, IL-6

## Abstract

Bioactivity-guided fractionation for the stems of leaves of *Larrea nitida* Cav., using interleukin-6 (IL-6) inhibitory assay in human mast cells (HMC-1), led to the isolation of three new compounds with an unprecedented skeleton in nature (**1**–**3**) and three known compounds (**4**–**6**). Their structures were elucidated through extensive spectroscopic analysis. The three new compounds were elucidated as two new spiroketones, nitidaones A (**1**), and B (**2**) and one new biphenyl analog, nitidaol (**3**). The known compounds were identified as nordihydroguaiaretic acid (**4**), 7,3′,4′-tri-*O*-methylquercetin (**5**) and ayanin (**6**). All the isolates were tested for their inhibitory activity against IL-6 production in HMC-1 cells. Of them, compounds **1**, **3**–**6** showed potent anti-inflammatory activity, with IC_50_ values of 12.8, 17.5, 14.9, 22.9, and 17.8 µM, respectively.

## 1. Introduction

The genus *Larrea* (Zygophyllaceae) has been used for ethnobotanical purposes by the native peoples of northwest, central, and southeast Argentina. This genus comprises five species seen in North and South America: *L. ameghinoi* Speg., *L. nitida* Cav., *L. divaricata* Cav., *L. mexicana* Moric., and *L. tridentata* (DC.) Coville [1,2]. Phytochemical studies on the genus *Larrea* reported the occurrence of flavonoids, lignans, naphthoquinones, saponins, and tannins [3,4]. These plants have been used for the treatment of cancer, inflammation, and menstrual pains [5,6,7]. Previous phytochemical investigations on *L. nitida* have reported the presence of flavonoids and lignans [8,9]. The biological activities of its extracts or individual ingredients included antifungal activity associated with Argentinean Andean propolis, antioxidant activity, and estrogenic activity [8,9,10]. As a part of our search for IL-6 production inhibitors from higher plants [11,12,13], *L. nitida* was selected for further isolation work due to IL-6 production inhibitory activity in the initial screening. Herein, the structural elucidation of three new compounds along with three known compounds and their IL-6 production inhibitory activity are described.

## 2. Results and Discussion

Air-dried stems and leaves of *L. nitida* (52 g) were extracted with MeOH, and the extract was subjected to bioactivity-guided isolation using diverse chromatography to afford two new spiroketones (**1**, **2**), one new biphenyl analog (**3**), and three known compounds. The known compounds were identified as nordihydroguaiaretic acid [14] (**4**), 7,3′,4′-tri-*O*-methylquercetin [15] (**5**), and ayanin [16] (**6**).

Compound **1** was obtained as an amorphous solid, and its molecular formula was deduced as C_21_H_26_O_4_ by the protonated ion peak [M + H]^+^ at *m*/*z* 343.1918 (calcd. for C_21_H_27_O_4_, *m*/*z* 343.1909). The ^1^H NMR disclosed the presence of a *para*-substituted benzene ring at δ_H_ 7.28 (2H, d, *J* = 9.0 Hz, H-2′,6′), and 6.89 (2H, d, *J* = 9.0 Hz, H-3′,5′), four olefinic protons at δ_H_ 7.19 (1H, dd, *J* = 10.0, 3.0 Hz, H-3), 7.10 (1H, dd, *J* = 10.0, 3.0 Hz, H-5), 6.32 (1H, dd, *J* = 10.0, 1.9 Hz, H-6) and 6.29 (1H, dd, *J* = 10.0, 1.9 Hz, H-2), an acetyl group at δ_H_ 2.00 (3H, s, OCOCH_3_), and a methoxy group at δ_H_ 3.76 (3H, s, OCH_3_) (Table 1).

From the HMBC NMR spectrum, the correlations between both H-3 and H-5 and the carbonyl carbon at δc 188.6 (C-1) and between both H-2 and H-6 and the quaternary carbon at δc 50.5 (C-4) suggested a quinoid-type skeleton. The 4-methoxyphenyl group was suggested by the HMBC correlations between one quaternary aromatic carbon at δc 133.4 (C-1′) and H-3′,5′ and between another quaternary aromatic carbon at δc 160.6 (C-4′) and both methoxy protons (δ_H_ 3.76) and two aromatic protons (H-2′,6′). A 2,3-dimethylbutoxy group was confirmed by the ^1^H and COSY NMR signals at δ_H_ 4.03 (1H, dd, *J* = 11.1, 6.9 Hz, H-10a), 3.85 (1H, dd, *J* = 11.1, 6.8 Hz, H-10b), 2.29 (1H, dd, *J* = 13.9, 2.4 Hz, H-7a), 1.95 (1H, dd, *J* = 13.9, 7.6 Hz, H-7b), 1.77 (1H, m, H-9), 1.45 (1H, m, H-8), 0.93 (3H, d, *J* = 7.0 Hz, H-11) and 0.89 (3H, d, *J* = 7.0 Hz, H-12). The COSY NMR spectrum showed correlations between H-11 and H-8, between H-12 and H-9 and sequential correlations from H-7 to H-10.

**Figure 1:**
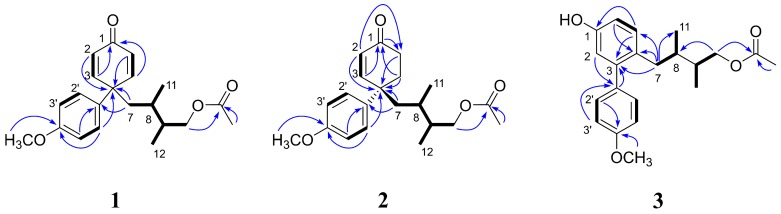
Key ^1^H, ^1^H-COSY (**bold line**) and HMBC (**blue arrow**) correlations of compounds **1**–**3**.

The link between these groups was confirmed by the HMBC correlations (Figure 1). The correlations between 4-methoxyphenyl protons (H-2′,6′) and C-4 (δ_C_ 50.5) and between protons (H-7a and 7b) on a 2,3-dimethylbutoxy group and both C-4 and C-1′ enabled us to put these groups together via a spiral carbon at C-4, as depicted in Figure 1. The acetyl group was affixed to C-10 from the observed correlations between δ_C_ 173.1 and both H-10 and methyl protons (δ_H_ 2.00). Therefore, the structure of compound **1** was determined to be 4-(1-(4-methoxyphenyl)-4-oxocyclohexa-2,5-dienyl)-2,3-dimethylbutyl acetate, named nitidaone A.

A protonated molecular ion [M + H]^+^ at *m*/*z* 345.2069 (calcd. for C_21_H_29_O_4_, 345.2066) of compound **2** suggested its molecular formula as C_21_H_28_O_4_. The ^1^H NMR data of **2** (Table 1) were similar to those of **1** except for the absence of two olefinic protons. Instead of olefinic protons, methylene signals at δ_H_ 2.40–2.29 (2H) and δ_H_ 2.24–2.14 (2H) were observed in the spectrum of **2**, assignable to H-5 and H-6. Furthermore, the HMBC correlations of H-7 with C-4 (δ_C_ 44.7) and C-1′ (δ_C_ 137.0) and H-2′,6′ with C-4 confirmed its spiral skeleton (Figure 1). Taken together with all data, this compound was elucidated as 4-(1-(4-methoxyphenyl)-4-oxocyclohex-2-enyl)-2,3-dimethylbutyl acetate, named nitidaone B.

**Figure 2:**
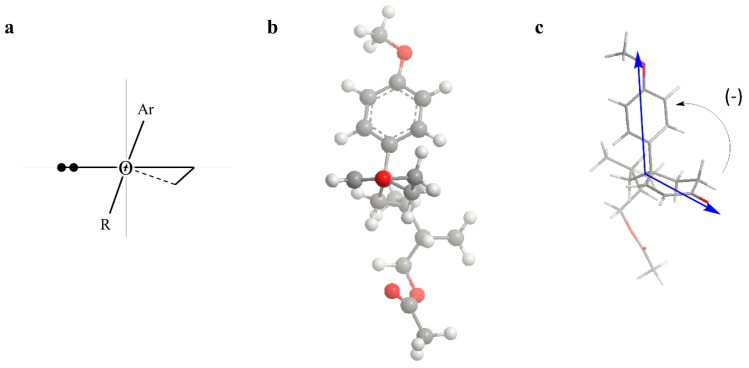
2-Cyclohexenone conformations: (**a**) Half-chair conformation with a pseudo-axial aromatic group giving a negative Cotton effect at 340 nm; (**b**) half-chair conformation with a pseudo-axial aromatic group of (4*R*)-**2**; (**c**) negative exciton-split Cotton effect of (4*R*)-**2**.

It has been reported that 2-cyclohexenones empirically give a negative Cotton effect (n→π^*^) in the 340 nm region if the ring has a half-chair conformation with a pseudo-axial disposition of an aryl group (Figure 2a) [17]. From a negative Cotton effect at 339 nm in the CD spectrum of **2**, it was affirmed that the cyclohexenone ring of this compound prefers the half-chair conformation with a pseudo-axial aromatic group at room temperature (Figure 2b). Therefore, the absolute configuration of C-4 was established to be *R*.

The exciton splitting theory supported its *R* configuration. From the UV and CD spectrum, the negative exciton-split Cotton effect at 220 nm was observed due to the π→π^*^ intramolecular charge-transfer transition of a 4-methoxyphenyl chromophore and a 2-cyclohexenone chromophore [18,19]. The negative exciton-split Cotton effect indicated that the two electric transition moments of these chromophores were rotated in a counterclockwise direction, as shown in Figure 2c.

The ^1^H and COSY NMR data of compound **3** displayed an 1,2,4-substituted benzene ring at δ_H_ 7.01 (1H, d, *J* = 8.3 Hz, H-5), 6.67 (1H, dd, *J* = 8.3, 2.7 Hz, H-6) and 6.58 (1H, d, *J* = 2.7 Hz, H-2), an 1,4-disubstituted benzene ring at δ_H_ 7.16 (2H, d, *J* = 8.8 Hz, H-2′,6′) and 6.92 (2H, d, *J* = 8.8 Hz, H-3′,5′) and a 2,3-dimethylbutoxy group at δ_H_ 3.66 (2H, m, H-10), 2.78 (1H, dd, *J* = 13.6, 4.9 Hz, H-7a), 2.19 (1H, dd, *J* = 13.6, 9.7 Hz, H-7b), 1.55 (1H, m, H-9), 1.47 (1H, m, H-8), 0.74 (3H, d, *J* = 6.9 Hz, H-12), and 0.65 (3H, d, *J* = 6.9 Hz, H-11). Also, a methoxy group at δ_H_ 3.82 (3H, s, OCH_3_) and an acetyl group at δ_H_ 1.95 (3H, s, OCOCH_3_) was observed (Table 2).

From the HMBC NMR spectrum, the correlations between H-2 and two quaternary aromatic carbons at δc 156.4 (C-1) and 144.5 (C-3) and between H-6 and another quaternary carbon at δc 131.1 (C-4) confirmed a 1-hydroxy-3,4-disubstituted benzene ring. The 4-methoxyphenyl group was suggested by the HMBC correlations between one quaternary aromatic carbon at δc 136.0 (C-1′) and H-3′,5′ and between the other quaternary aromatic carbon at δc 160.2 (C-4′) and both methoxy protons (δ_H_ 3.82) and two aromatic protons (H-2′,6′). A 2,3-dimethylbutoxy group was assigned by the sequential COSY correlations from H-7 to H-11 and from H-10 to H-12 and the HMBC correlation between H-10 and C-8 (δ_C_ 37.8). The link of these substructures was confirmed by the observed HMBC correlations (Figure 1). The HMBC signals between H-2 and C-1′ and between H-2′,6′ and C-3 indicated that C-3 was connected to C-1′. The HMBC correlations between H-7 and C-3, C-4 and C-5 enabled to connect a 2,3-dimethylbutoxy group to C-4 position. The correlations of δ_C_ 173.1 with both H-10 and a methyl proton (δ_H_ 1.95) made it possible to link the acetyl group to C-10 position through an ester linkage (Figure 1). Therefore, the structure of this compound was determined to be 4-(5-hydroxy-4′-methoxybiphenyl-2-yl)-2,3-dimethylbutyl acetate, named nitidaol, as shown in Figure 1.

All the isolates were tested against IL-6 production inhibitory activity in the HMC-1 cells induced by PMA + A23187. Of these, compounds **1** and **3**–**6** were found to be active with IC_50_ 12.8, 17.5, 14.9, 22.9 and 17.8 µM, respectively (positive control, IC_50_ of montelukast, 8.7 µM), while compound **2** seemed inactive (IC_50_ > 25 µM).

## 3. Materials and Methods

### 3.1. General Experimental Procedures

Optical rotation was measured with a Jasco P2000 polarimeter (Jasco Corporation, Tokyo, Japan), and FT-IR spectra using a Jasco FT/IR-4200 (Jasco corporation, Japan). ECD and UV spectra were recorded with an Applied Photophysics Chirascan-plus CD spectrometer. ^1^H, ^13^C and 2D NMR spectra were obtained on a Varian 400 (Varian, Palo Alto, CA, USA)-400MHz. Waters Xevo G2 Q-TOF, (Waters, Milford, MA, USA) spectra were measured on a Q-TOF mass spectrometer. Semi-preparative high-performance liquid chromatography (HPLC) was performed on a Gilson 321 pump, Gilson 172 Diode Array Detector (Gilson, Middleton, WI, USA). YMC-pack Ph, 250 × 20 mm (YMC, Tokyo, Japan) and Luna 5u C18 column 250 × 10 nm (Phenomenex) as HPLC columns were used. MPLC was run on Isolera One (Biotage, Cardiff, UK). Solvents for HPLC were acetonitrile (MeCN) (HPLC grade) and methanol (HPLC grade), purchased from SK Chemical (Seoul, Korea). Water was purified using a MIlli-Q system (Millipore, Bedford, MA, USA). Column chromatography was performed on C-18 RP silica gel (Cosmosil, Kyoto, Japan) and Sephadex LH-20 (GE Healthcare, Stockholm, Sweden). TLC analysis was run on silica gel 60 F_254_ plates (Marck, Darmstadt, Germany). The spots were visualized by spraying with 10% aqueous H_2_SO_4_.

### 3.2. Plant Material

The stems and leaves of *L. nitida* Cav. were collected at Jarilla, Chile in 2007 and identified by Dr. Joongku Lee, Korea Research Institute of Bioscience and Biotechnology. A voucher specimen (access number FBM026-052) was deposited in the herbarium of the Korea Research Institute of Bioscience and Biotechnology, Daejeon, Republic of Korea.

### 3.3. Extraction and Isolation

Air-dried stems and leaves of *L. nitida* (52 g) were pulverized and extracted with MeOH to yield the crude extract (14 g). A portion of this extract (12 g) was subjected to a reverse-phase silica gel column chromatography eluting with a gradient of H_2_O–MeOH (90:10–0:100) to give 17 sub-fractions (LN01-17). Of these sub-fractions, LN 8 and 11 were found to inhibit IL-6 production (over 50% inhibition at 20 µg/mL) in HMC-1 cells stimulated by PMA + A23187 (Figure S6). LC8 (1 g) was fractionated into 14 sub-fractions using a reversed phase silica gel column chromatography with a gradient mixture of MeOH–H_2_O (25:75 to 100:0).

Further HPLC separation for LC8-9 (Phenomenex Luna C18, MeCN 40%) yielded nordihydroguiaretic acid (6.8 mg, *t*_R_ 38.04 min). LN11 (1.2 g) was further fractionated by medium-pressure liquid chromatography (Biotage), eluted with a gradient mixture of MeOH–H_2_O = 40:60–100:0), and then pooled into 16 sub-fractions (LN11-1 to LN11-16). Compound **1** (14.5 mg, *t*_R_ 84.73 min) was separated from LN11-10 (106 mg) using HPLC separation (Phenomenex Luna C18 250 × 10, 5 μm, 2 mL/min, 45% MeCN in H_2_O). LN11-12 (249 mg) was separated using an HPLC column (Phenomenex Luna C18 250 × 10, 5 μm, 2 mL/min) eluted with a gradient solvent system [45% in H2O (1 to 56 min), 55% in H2O (56.01 to 71 min), 80% in H2O (71.01 to 78 min), 100% MeCN (78.01 to 85 min)] to yield 3′,4′,7-trimethylquercetin (1.7 mg, tR 54.15 min), 3,7,3′-trimethylquercetin (1.6 mg, tR = 55.98 min), and 2 (5.1 mg, tR 78.00 min). From LN11-13 (118 mg), compound **3** (5.0 mg, *t*_R_ = 28.58 min) was purified using sequentially connected two columns [Luna C18 (250 × 10, 5 μm) + YMC-packed ODS-AM (150 × 10, 2 mL/min, 5 μm)] with isocratic elution of 70% MeCN in H_2_O.

*Nitidaone A* (**1**): white amorphous solid; ${\left[\alpha \right]}_{D}^{20}$ −42.1° (*c* 0.5, MeOH); UV (MeOH) λ_max_ (log ε) 256 (3.60), 272 (3.41) nm; ^1^H and ^13^C NMR data, see Table 1; HRESIMS *m*/*z* 343.1918 [M + H]^+^ (calcd. for C_21_H_26_O_4_, 343.1909).
*Nitidaone B* (**2**): white amorphous solid; ${\left[\alpha \right]}_{D}^{20}$ −53.9° (*c* 0.1, MeOH); UV (MeOH) λ_max_ (log ε) 208 (3.79), 212 (3.82) nm; CD (MeOH) λ_max_ (Δ*_ε_*) 208 (−0.05), 216 (0.08), 233 (−0.30), 339 (−0.04); ^1^H and ^13^C NMR data, see Table 1; HRESIMS *m*/*z* 345.2069 [M + H]^+^ (calcd. for C_21_H_28_O_4_, 345.2066).
*Nitidaol* (**3**): white amorphous solid; ${\left[\alpha \right]}_{D}^{20}$ −4.94° (*c* 0.2, MeOH); UV (MeOH) λ_max_ (log ε) 257 (3.63), 281 (3.60) nm; ^1^H and ^13^C NMR data, see Table 2; HRESIMS *m*/*z* 343.1913 [M + H]^+^ (calcd. for C_21_H_26_O_4_, 343.1909).

### 3.4. Interleukin-6 Determination

Cells were seeded at 1 × 10^6^/mL per well in 24-well tissue culture plates and pretreated with the indicated concentration of compounds for 30 min before stimulation by PMA (50 nM) + A23187 (1 µM). After 24 h, the supernatant was decanted into a new micro-centrifuge tube, and the amount of interleukin-6 (IL-6) was determined using an enzyme-linked immunosorbent assay (ELISA) kit according to the procedures described by the manufacturer (BD Biosciences, San Jose, CA, USA). All subsequent steps took place at room temperature, and all standards and samples were assayed in duplicate [13].

## 4. Conclusions

Two new spiroketones (**1**, **2**) and one new biphenyl analog (**3**), together with three known compounds, were isolated from the stems and leaves of *L. nitida*. Three new compounds (**1**–**3**) have unprecedented skeletons in nature. The absolute configuration of C-4 (compound **2**) was demonstrated by ECD analysis, but that of C-8 and C-9 (compounds **1**–**3**) could not be identified. All the isolates were tested against IL-6 production inhibitory activity in the HMC-1 cells induced by PMA+A23187. It was found that nitidaone A (**1**), nitidaol (**3**), nordihydroguaiaretic acid (**4**), 7,3′,4′-trimethylquercetin (**5**), and 3,7,3′-trimethylquercetin (**6**) remarkably downregulated the PMA+A23187-induced synthesis of interleukin-6 (IL-6) in HMC-1 cells without cytotoxicity.

## Figures and Tables

**Table 1:** The ^1^H and ^13^C NMR data of compounds **1** and **2** in methanol-*d*_4_.

	Nitidaone A (1) ^a^			Nitidaone B (2) ^a^		
Position	δ_C_	Type	δ_H_	δ_C_	Type	δ_H_ (*J* in Hz)
1	188.6	C		202.0	C	
2	128.5	CH	6.29, dd (10.0, 1.9)	129.3	CH	6.06, d (10.3)
3	158.4	CH	7.19, dd (10.0, 3.0)	159.8	CH	7.38, dd (10.3, 1.5)
4	50.5	C		44.7	C	
5	157.9	CH	7.10, dd (10.0, 3.0)	36.3	CH	2.29–2.40, overlap; 2.14–2.24, overlap
6	129.2	CH	6.32, dd (10.0,1.9)	35.6	CH	2.29-2.40, overlap; 2.14–2.24, overlap
7	43.2	CH_2_	2.29, dd (13.9, 2.4); 1.95, dd (13.9, 7.6)	46.7	CH_2_	1.94, d (11.7); 1.61–1.67, overlap
8	32.7	CH	1.45, m	31.9	CH	1.62–1.68, overlap
9	39.8	CH	1.77, m	40.1	CH	1.63–1.69, overlap
10	68.1	CH_2_	4.03, dd (11.1, 6.9); 3.85, dd (11.1, 6.8)	68.2	CH_2_	3.90, dd (11.0, 6.6); 3.78, dd (11.0, 6.7)
11	19.8	CH_3_	0.93, d (7.0)	19.2	CH_3_	0.71, d (6.4)
12	13.9	CH_3_	0.89, d (7.0)	13.7	CH_3_	0.83, d (6.9)
1′	133.4	C		137.0	C	
2′, 6′	129.0	CH	7.28, d (9.0)	129.3	CH	7.29, d (8.9)
3′, 5′	115.5	CH	6.89, d (9.0)	115.1	CH	6.90, d (8.9)
4′	160.6	C		160.0	C	
4′-OCH_3_	55.9	CH_3_	3.76, s	55.8	CH_3_	3.78, s
COCH_3_	21.0	CH_3_	2.00, s	21.0	CH_3_	1.98, s
OCO	173.1	C		173.0	C	

^a 1^H and ^13^C NMR were measured at 400 and 100 MHz, respectively.

**Table 2:** The ^1^H and ^13^C NMR data of compound **3** in methanol-*d*_4_

	Nitidaol (3) ^a^		
Position	δ_C_	Type	δ_H_ (*J* in Hz)
1	156.4	C	
2	118.2	CH	6.58, d (2.7)
3	144.5	C	
4	131.1	C	
5	132.6	CH	7.01, d (8.3)
6	114.9	CH	6.67, dd (8.3, 2.7)
7	36.8	CH_2_	2.78 dd (13.6, 4.9)
			2.19, dd (13.6, 9.7)
8	37.8	CH	1.47, m
9	38.2	CH	1.55, m
10	68.7	CH_2_	3.66, m
11	16.8	CH_3_	0.65, d (6.9)
12	14.1	CH_3_	0.74, d (6.9)
1′	136.0	C	
2′, 6′	131.4	CH	7.16, d (8.8)
3′, 5′	114.7	CH	6.92, d (8.8)
4′	160.2	C	
4′-OCH_3_	55.8	CH_3_	3.82, s
COCH_3_	21.0	CH_3_	1.95, s
OCO	173.1	C	

^a 1^H and ^13^C NMR were measured at 400 and 100 MHz, respectively.

## Supplementary Materials

The supplementary materials are available online.
