# Supplementary material for: Spiroketones and a Biphenyl Analog from Stems and Leaves of Larrea nitida and Their Inhibitory Activity against IL-6 Production
Source: Molecules. 2018 Jan 31;23(2):302. doi: 10.3390/molecules23020302 (PMC6017194; doi:10.3390/molecules23020302)

SUPPORTING INFORMATION

Spiroketones and a biphenyl analog from stems and leaves of *Larrea nitida* and their inhibitory activity against IL-6 production

Jongmin Ahn [1,#], Yihua Pei [2,#], Hee-Sung Chae [2], Seong-Hwan Kim [1], Young-Mi Kim [2], Young Hee Choi [2], Joongku Lee [3], Sei-Ryang Oh [4], Minsun Chang [5], Yun Seon Song [6], Roberto Rodriguez [7], Dong-Chan Oh [1], Jinwoong Kim [1], Sangho Choi [8], Sang Hoon Joo [9] and Young-Won Chin [2,*]

[1] College of Pharmacy and Research Institute of Pharmaceutical Sciences, Seoul National University, Seoul 08826, Republic of Korea
[2] College of Pharmacy and Integrated Research Institute for Drug Development, Dongguk University-Seoul, Gyeonggi-do 10326, Republic of Korea
[3] Department of Environment and Forest Resources, College of Agriculture and Life Sciences, Chungnam National University, Daejeon 34134, Republic of Korea
[4] Natural Medicine Research Center, KRIBB, ChungBuk 363-883, Republic of Korea
[5] Department of Biological Sciences, College of Science, Sookmyung Women's University, Seoul 04310, Korea
[6] College of Pharmacy, Sookmyung Women's University, Seoul 04310, Republic of Korea
[7] Department of Botany, University of Concepcion, Casilla 160C, Concepcion, Chile
[8] International Biological Material Research Center, KRIBB, Daejeon 34141, Republic of Korea
[9] College of Pharmacy, Daegu Catholic University, Gyeongbuk 38430, Republic of Korea

# Authors equally contribute to this work.
* Correspondence: f2744@dongguk.edu (Y.-W. Chin); Tel.: +82-31-961-5218

Contents

Figure S 1. The ^{1}H NMR (400 MHz) and ^{13}C (100 MHz) spectra spectra of **1** in methanol-d_4

Figure S 2. The HSQC spectrum of **1** in methanol-d_4

Figure S 3. The HMBC spectrum of **1** in methanol-d_4

Figure S 4. The COSY spectrum of **1** in methanol-d_4

Figure S 5. The ^{1}H NMR (400 MHz) and ^{13}C (100 MHz) spectra spectra of **2** in methanol-d_4

Figure S 6. The HSQC spectrum of **2** in methanol-d_4

Figure S 7. The HMBC spectrum of **2** in methanol-d_4

Figure S 8. The COSY spectrum of **2** in methanol-d_4

Figure S 9. The CD and UV spectra of **2**

Figure S 10. The ^{1}H NMR (400 MHz) and ^{13}C (100 MHz) spectra spectra of **3** in methanol-d_4

Figure S 11. The HSQC spectrum of **3** in methanol-d_4

Figure S 12. The HMBC spectrum of **3** in methanol-d_4

Figure S 13. The COSY spectrum of **3** in methanol-d_4

Figure S 1. The ^{1}H NMR (400 MHz) and ^{13}C (100 MHz) spectra spectra of **1** in methanol-d_4

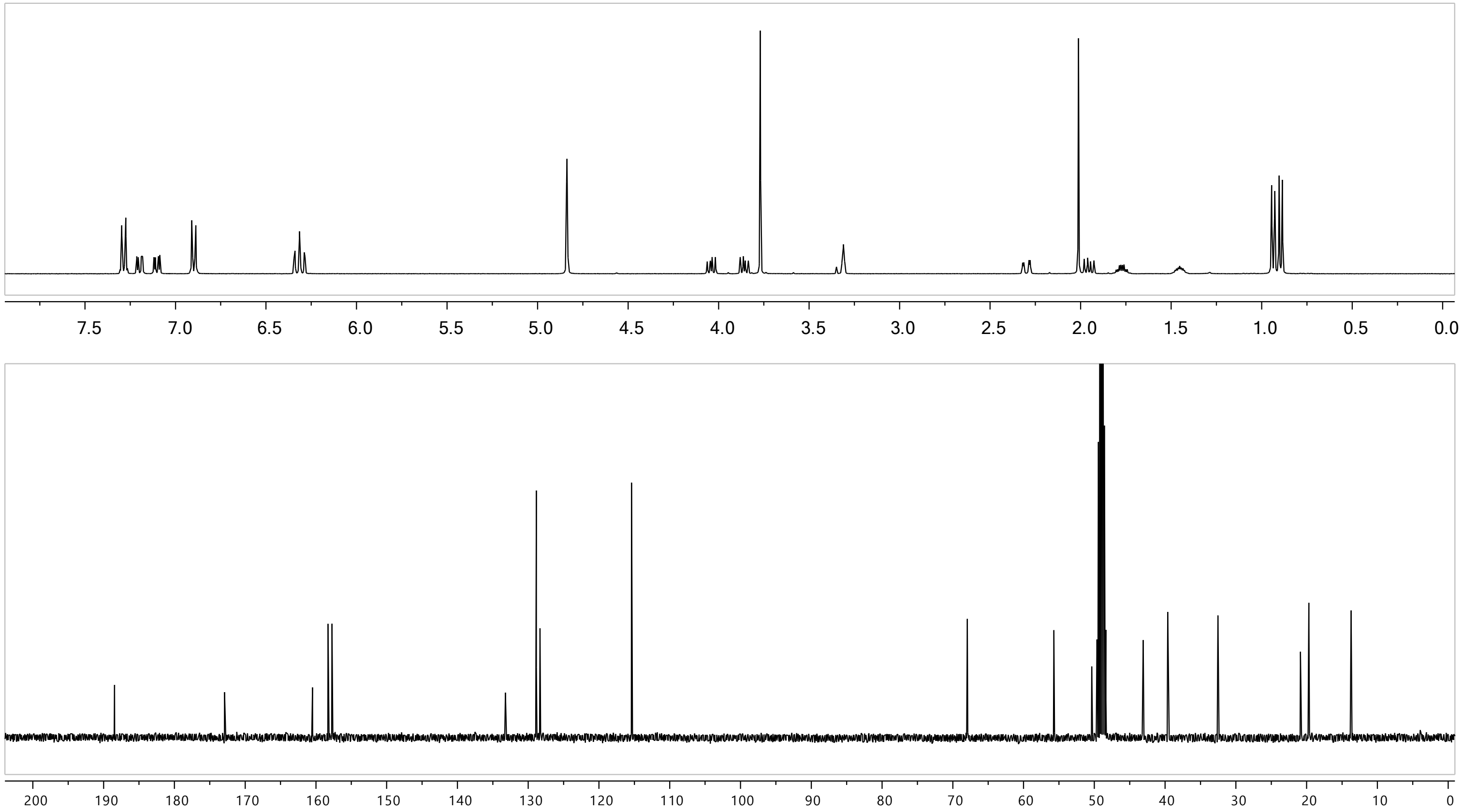

Figure S 2. The HSQC spectrum of **1** in methanol-d_4

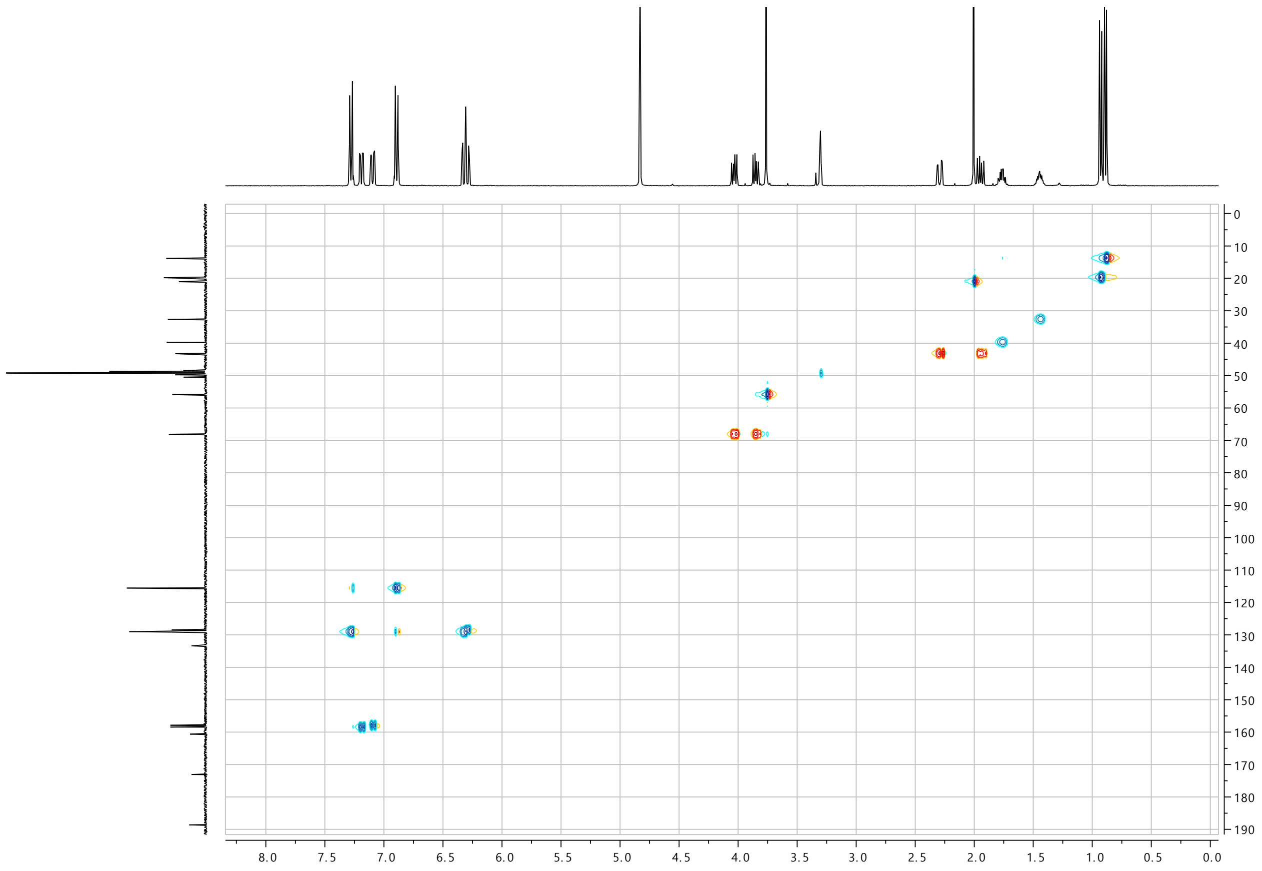

Figure S 3. The HMBC spectrum of **1** in methanol-d_4

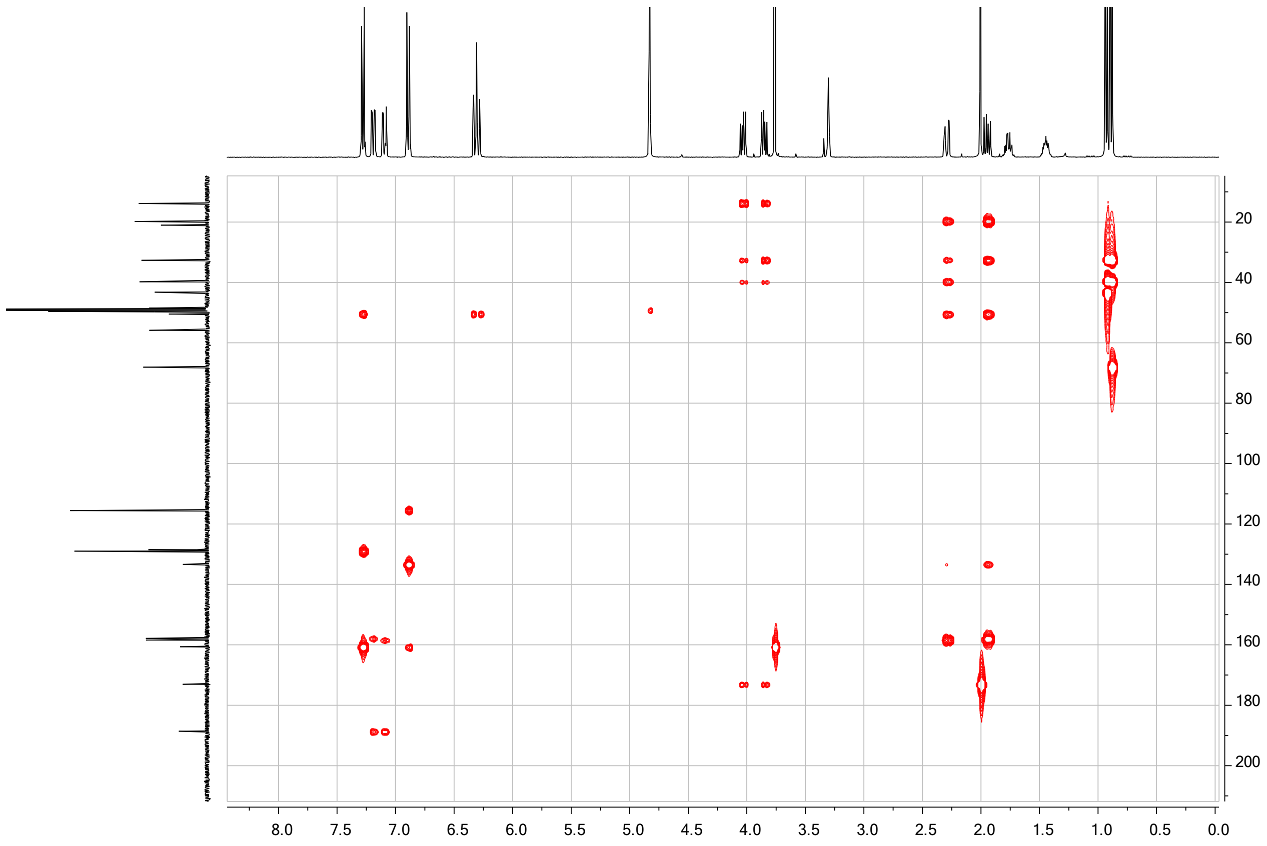

Figure S 4. The COSY spectrum of **1** in methanol-d_4

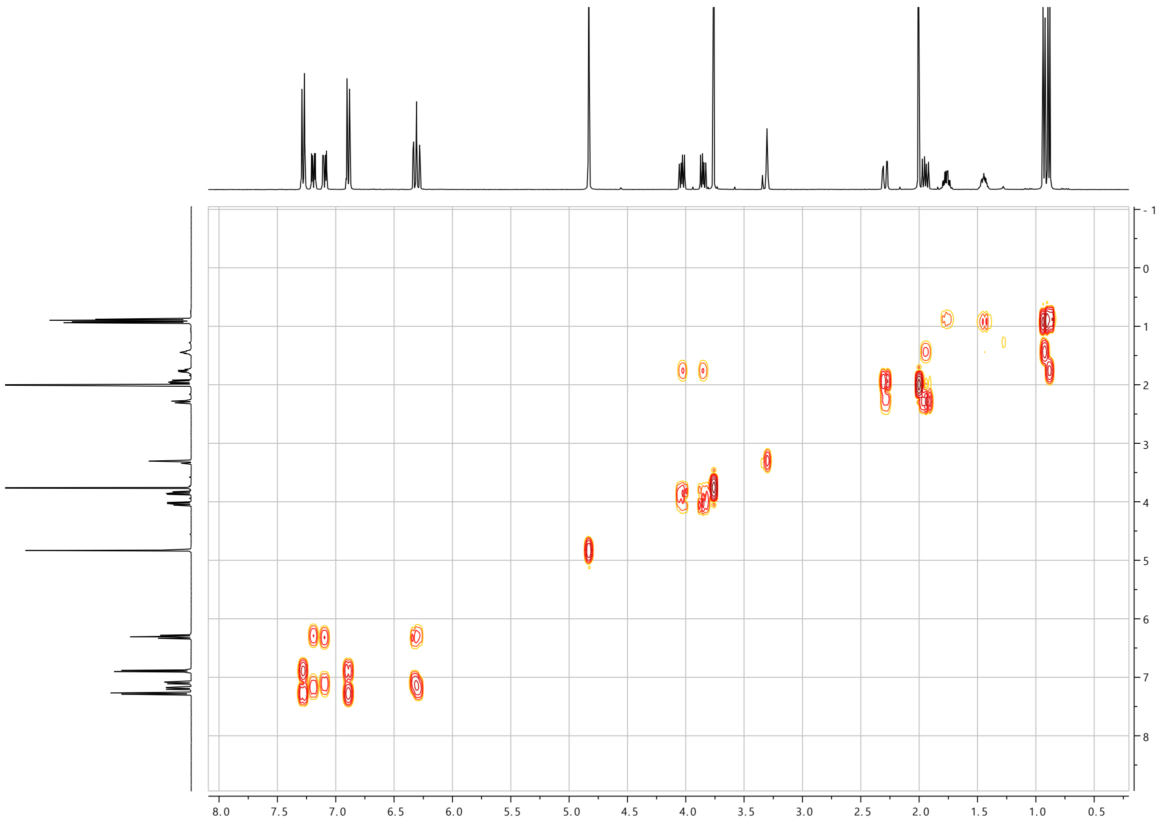

Figure S 5. The ^{1}H NMR (400 MHz) and ^{13}C (100 MHz) spectra spectra of **2** in methanol-d_4

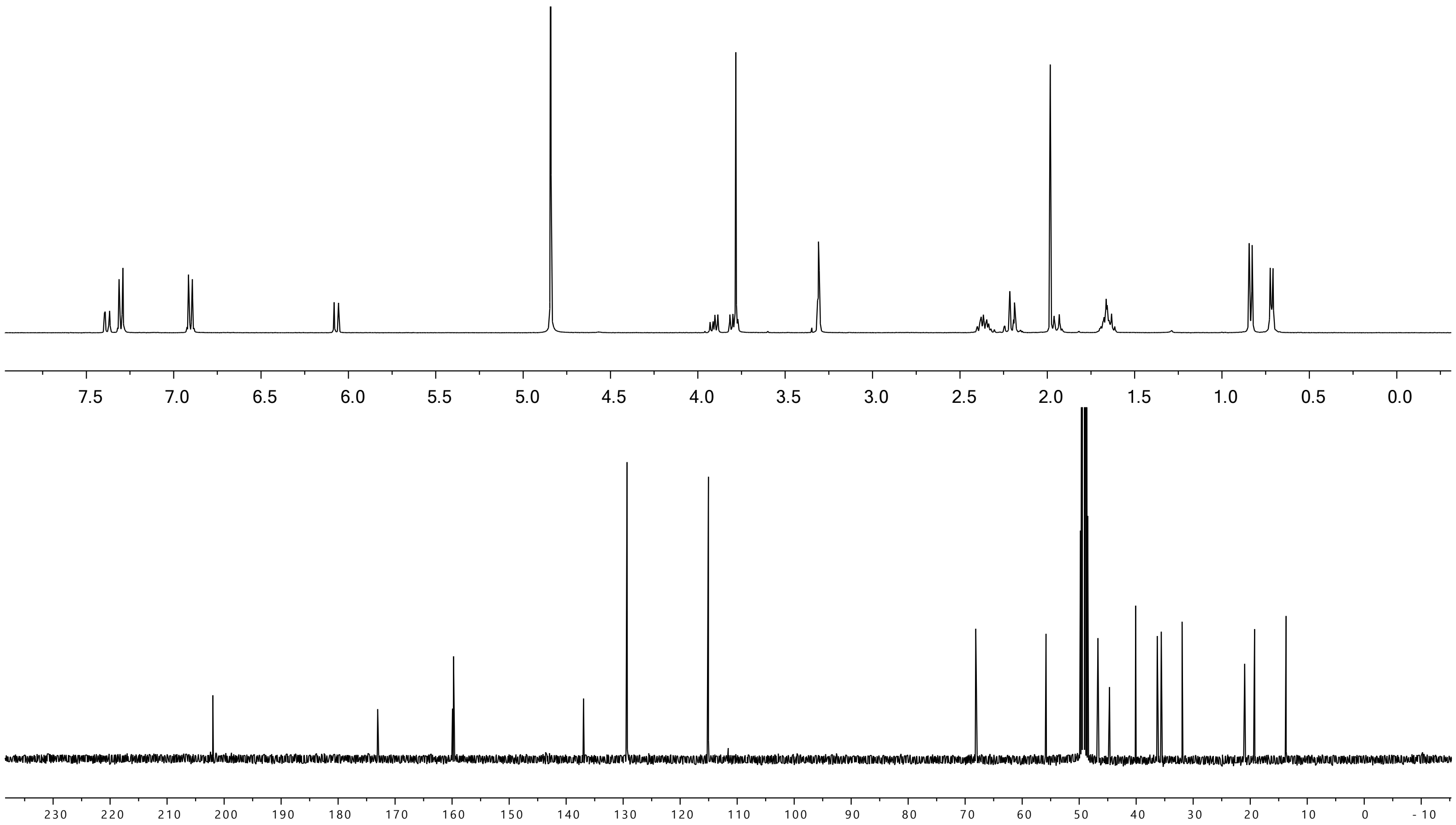

Figure S 6. The HSQC spectrum of **2** in methanol-d_4

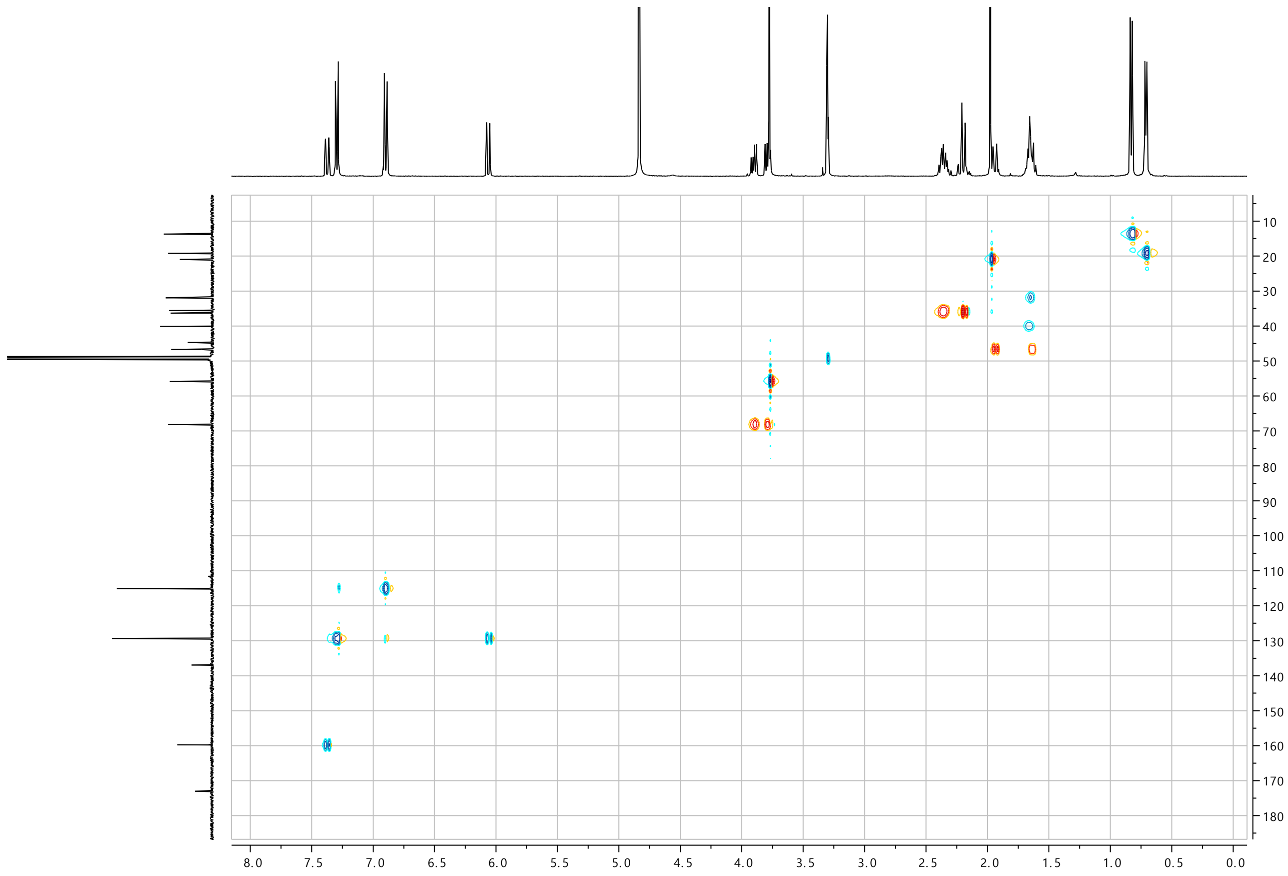

Figure S 7. The HMBC spectrum of **2** in methanol-d_4

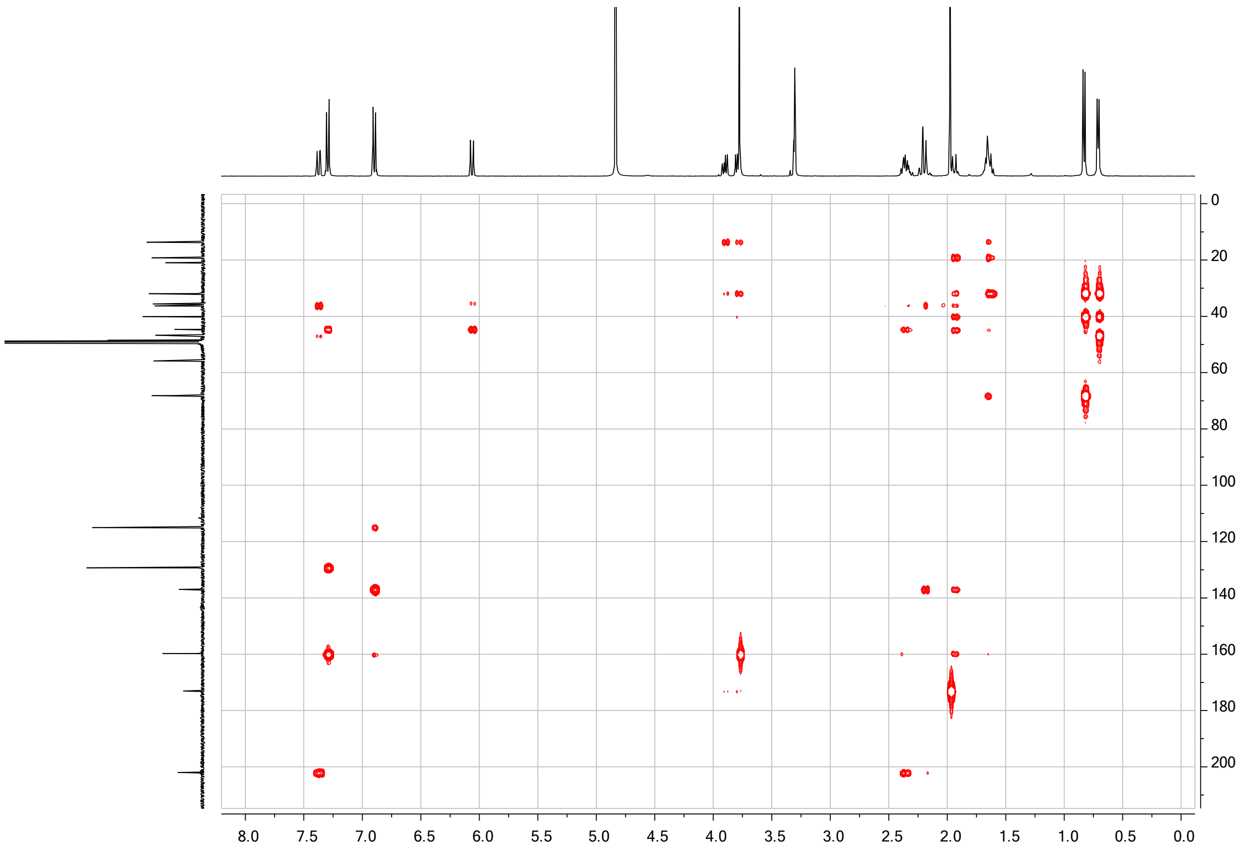

Figure S 8. The COSY spectrum of **2** in methanol-d_4

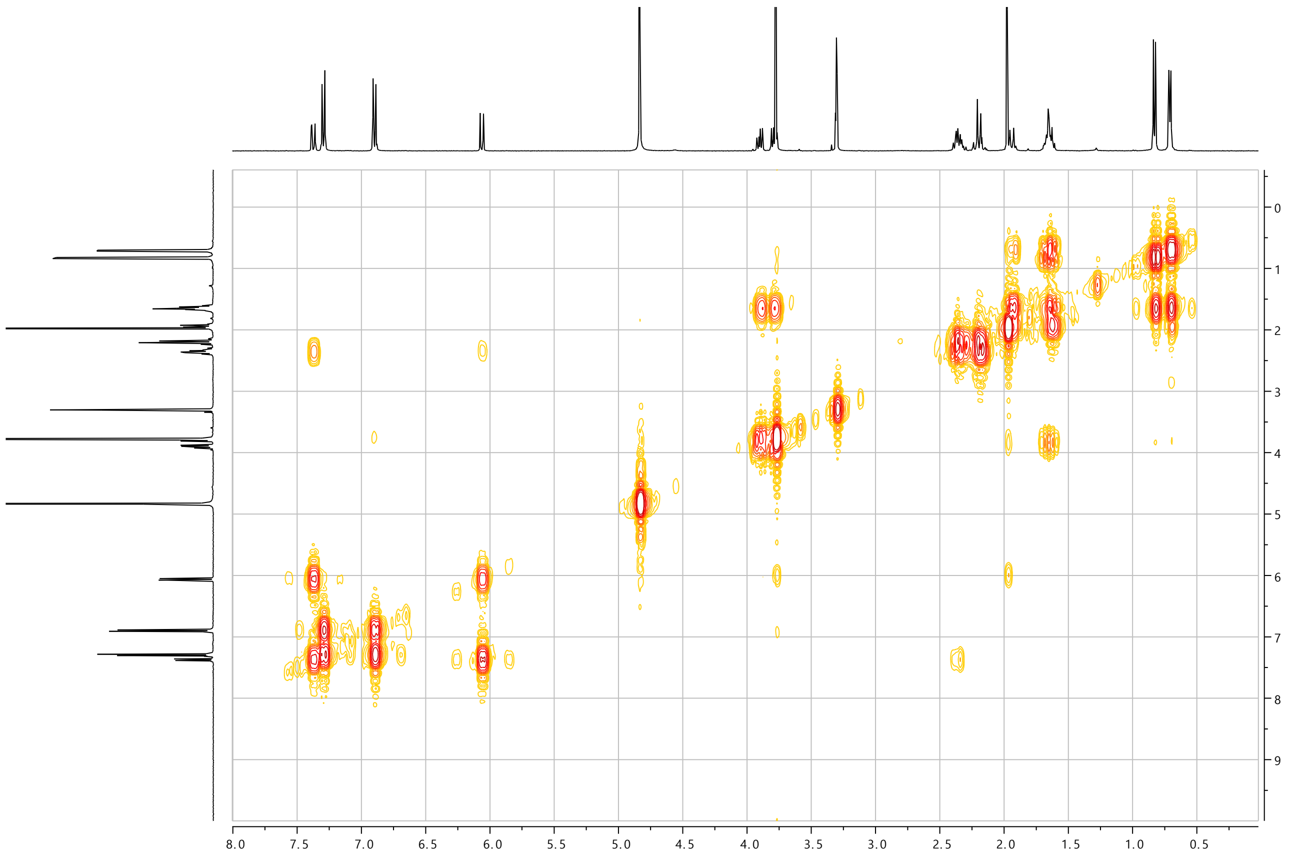

Figure S 9. The CD and UV spectra of **2**

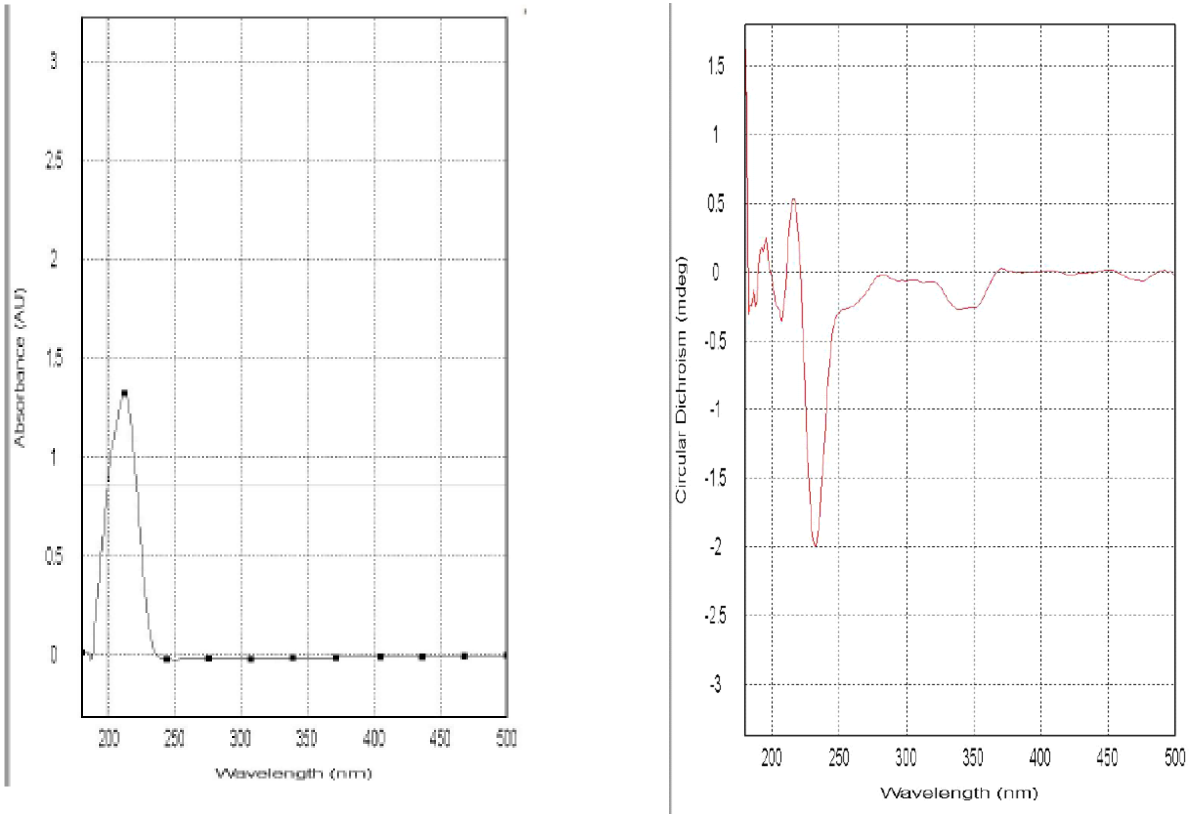

Figure S 10. The ^{1}H NMR (400 MHz) and ^{13}C (100 MHz) spectra spectra of **3** in methanol-d_4

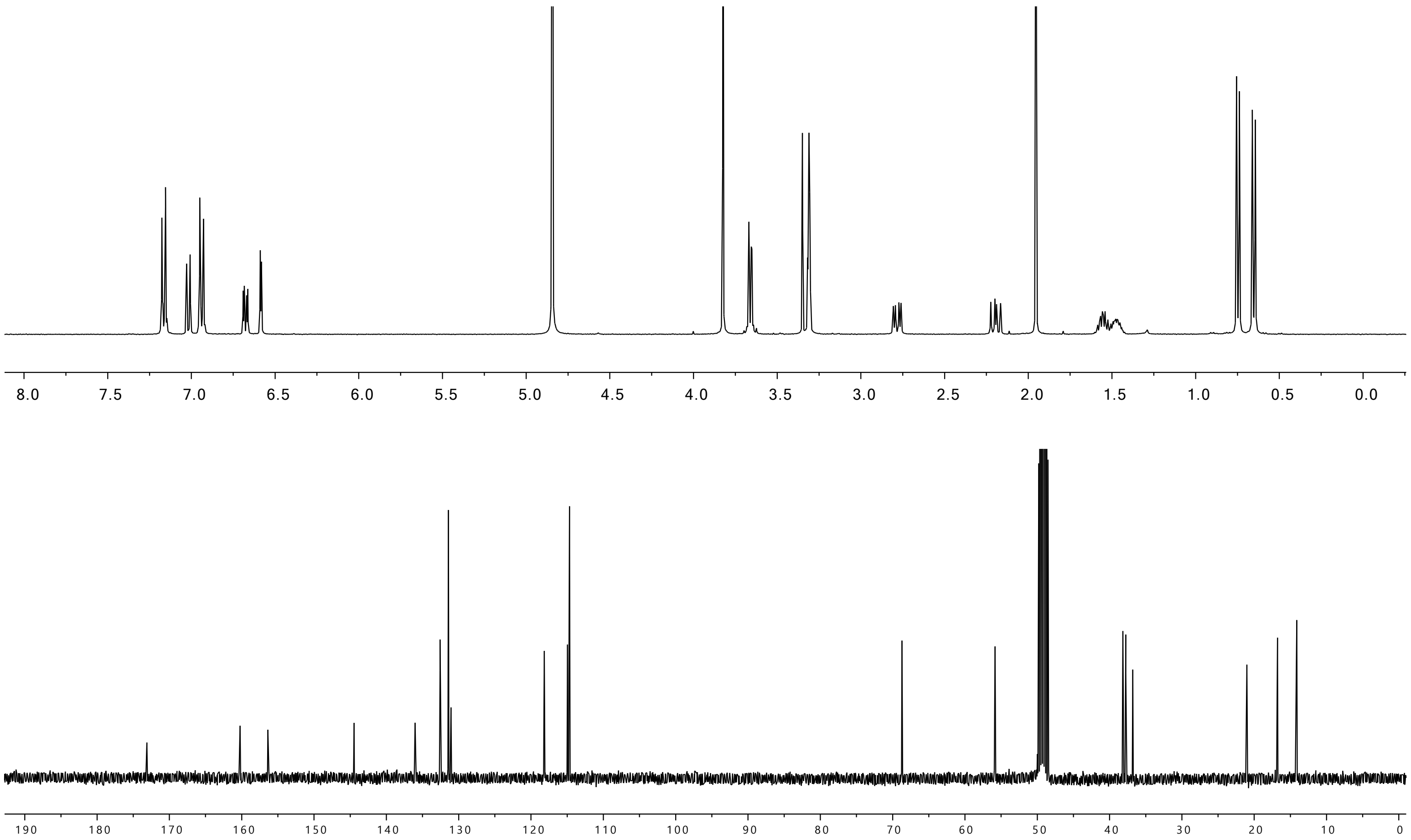

Figure S 11. The HSQC spectrum of **3** in methanol-d_4

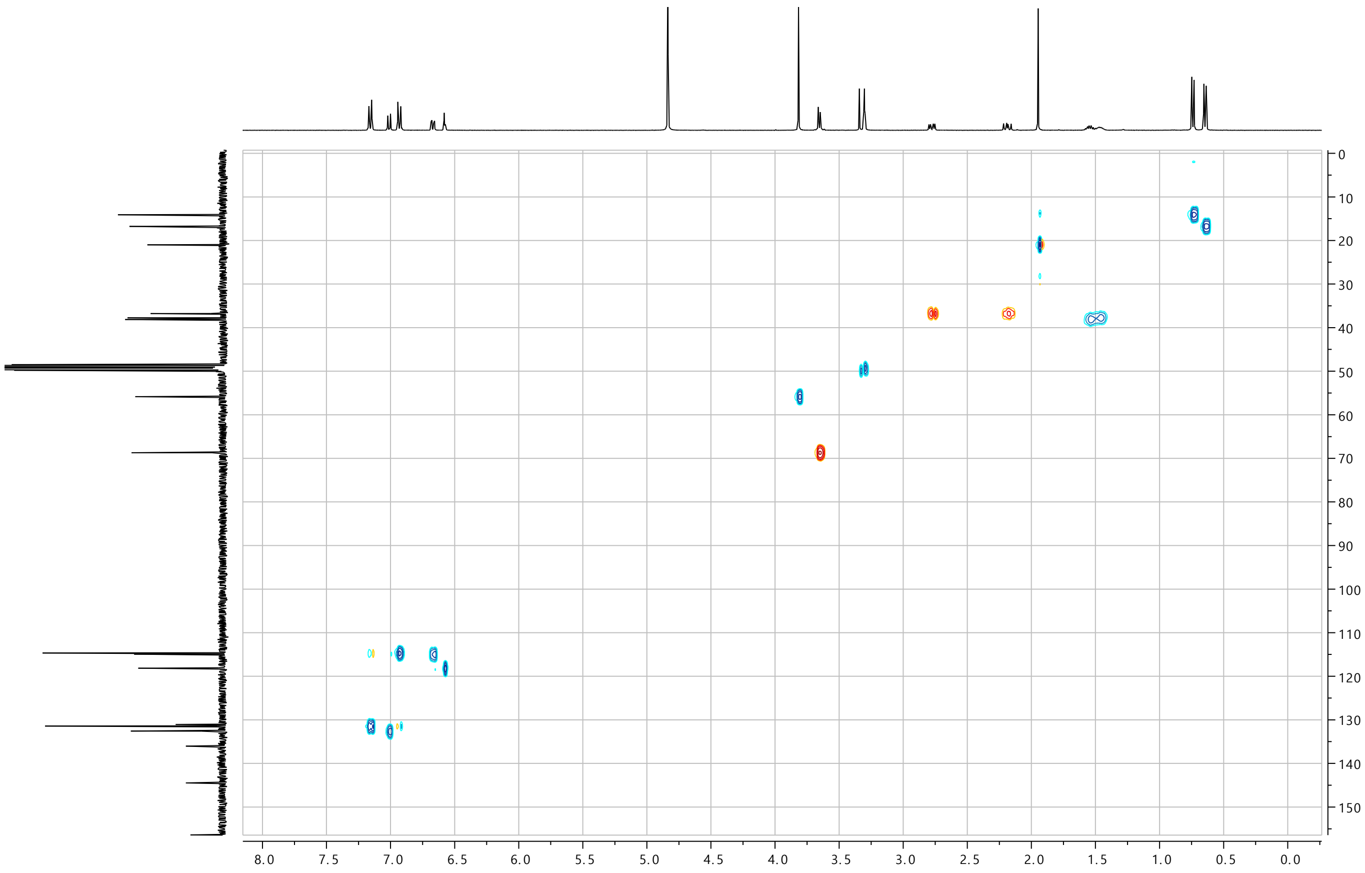

Figure S 12. The HMBC spectrum of **3** in methanol-d_4

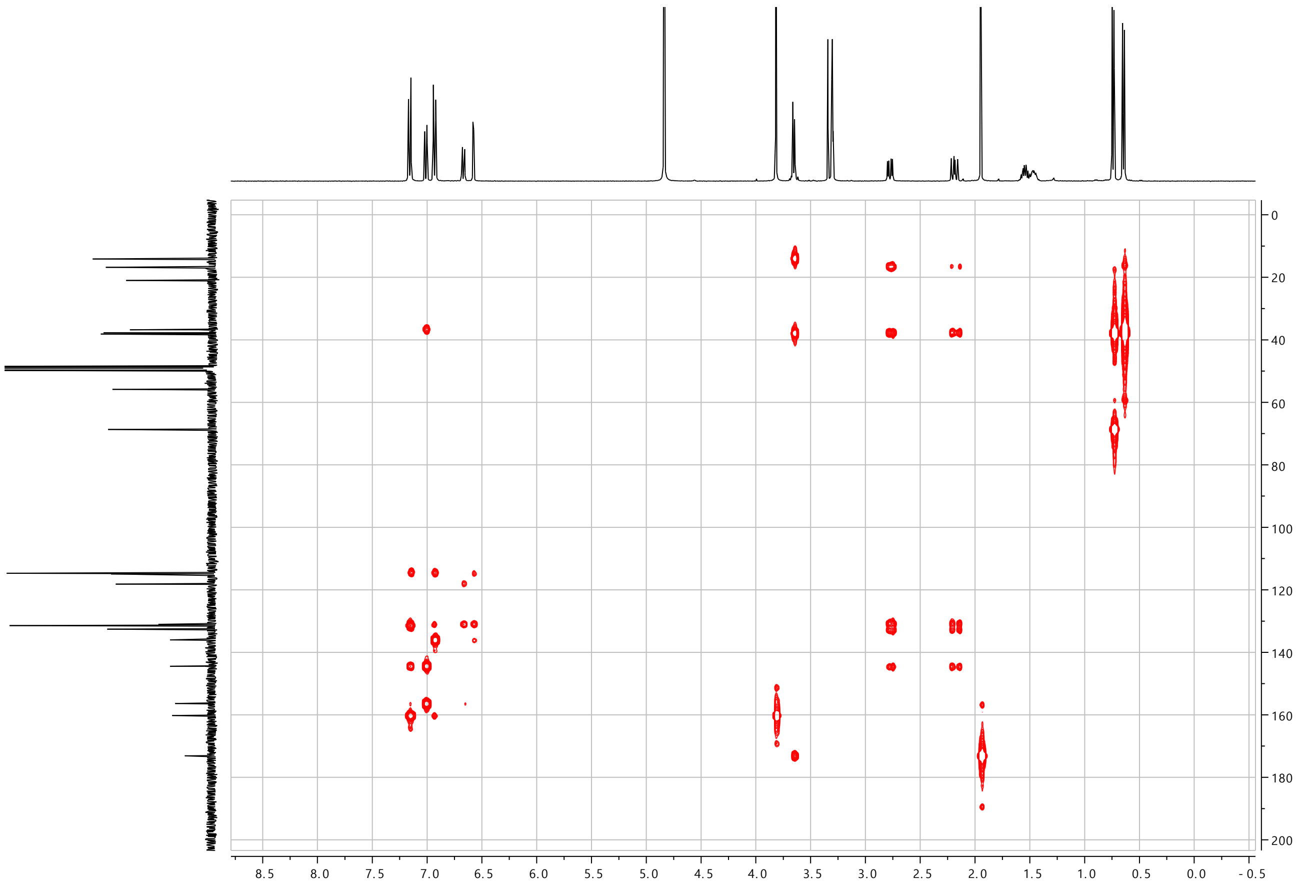

Figure S 13. The COSY spectrum of **3** in methanol-d_4

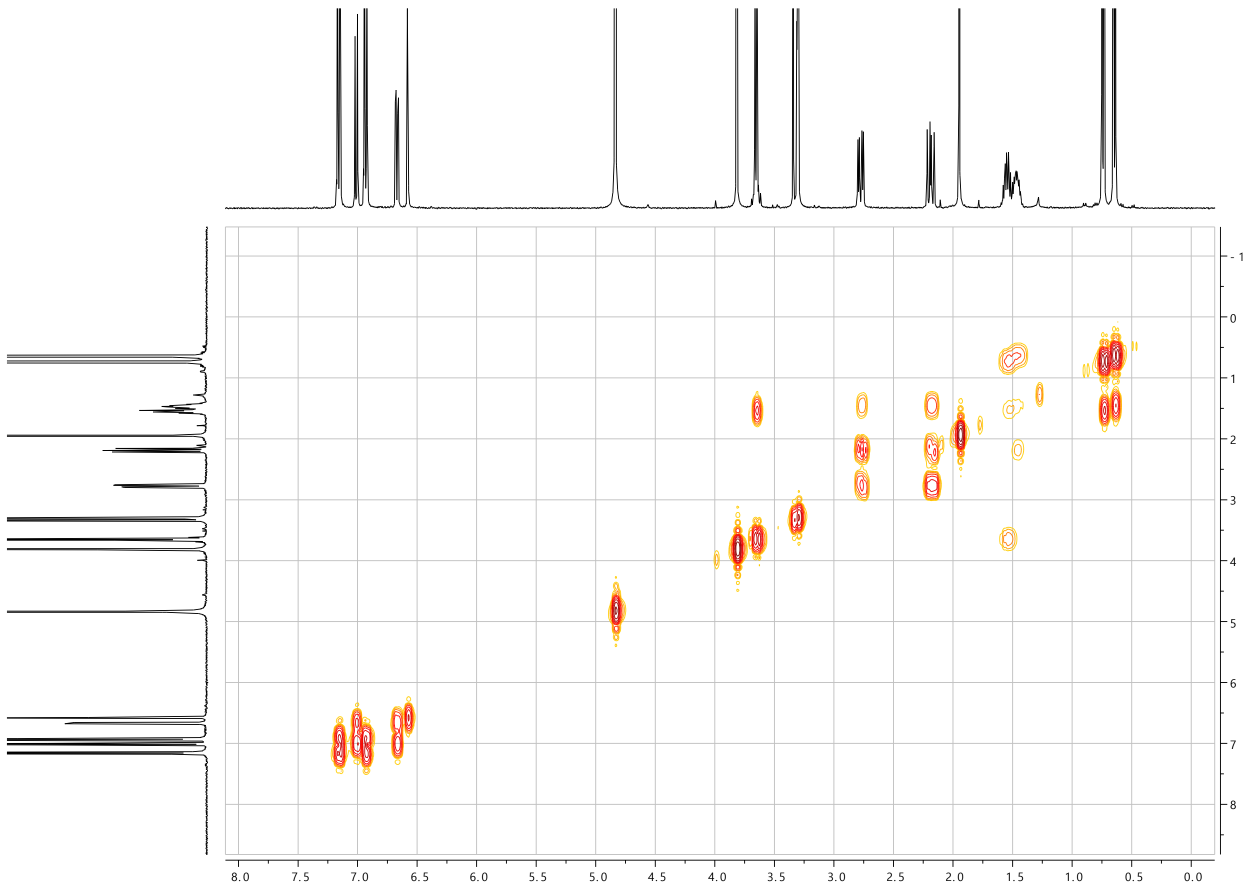

Supplement: Supplementary file 1 [file molecules-23-00302-s001.pdf]
